# Latrine Availability and Associated Factors among Religious Institutions in Northern Ethiopia, 2018

**DOI:** 10.1155/2020/6498412

**Published:** 2020-10-28

**Authors:** Mulugeta Woldu Abrha, Kiros Demoz Ghebremedhin, Tesfay Teklemariam Weldeslasie

**Affiliations:** Tigray Health Research Institute, Mekelle, Ethiopia

## Abstract

**Background:**

Religious institutions found in the community not only uphold belief and cultural values but can also act as a force for positive change and development. Improved sanitation and hygiene are crucial in these institutions to decrease preventable infections due to unsanitary conditions. However, there are no studies among religious institutions on availability of latrines. Therefore, this study was conducted to assess latrine availability and associated factors among religious institutions in the Tigray Region, Ethiopia.

**Method:**

An institution-based cross-sectional study design was conducted in the Tigray Region, Northern Ethiopia. Multistage sampling was used to sample 385 religious institutions. Data were collected using a pretested, structured questionnaire and observation checklist. Logistic regression was fitted, and an odds ratio with 95% confidence interval (CI) with *p* value less than 0.05 was used to determine the predictors of latrine availability. Analysis was carried out using the SPSS 20^TM^ software package.

**Results:**

In this study, latrine availability was 32.8%. It was significantly affected by currently saved money towards having a latrine (adjusted odds ratio (AOR): 0.32, 95% confidence interval (CI) [0.25, 0.42]), any messages seen, heard, or received on sanitation and hygiene (AOR: 0.43, 95% CI [0.38, 0.51]), and the place where messages were seen, heard, or received (AOR: 2.95, 95% CI [1.11, 5.55]).

**Conclusion:**

Latrine availability was very low when compared to the national target of 100% among religious institutions and was affected by the currently saved money towards having a latrine, any messages seen, heard, or received on sanitation and hygiene, and the place where the messages were received. Information regarding latrine availability should be provided to the community visiting religious institutions through available channels and promotion of practical models.

## 1. Background

Lack of sanitation is a serious health risk, affecting billions of people around the world, particularly the poor and disadvantaged. It also contributes to stunting and impaired cognitive function and impacts on well-being through school attendance, anxiety, and safety with lifelong consequences, especially for women and girls [1, 2]. Lack of sanitation facilities compels people to practice open defecation, which increases the risk of disease transmission and perpetuates a vicious cycle of disease and poverty. The countries where open defection is most widespread have the highest number of deaths of children aged under 5 years as well as the highest levels of malnutrition and poverty and big disparities of wealth [3]. The disease burden associated with poor water, sanitation, and hygiene is estimated to account for 4.0% of all deaths and 5.7% of the total disease burden in disability-adjusted life year (DALY) worldwide, principally through diarrheal diseases, schistosomiasis, trachoma, ascariasis, trichuriasis, and hookworm infection [4]. About 1.8 million people die every year due to diarrheal diseases, and children under the age of 5 account for 90% of diarrheal deaths. Moreover, 88% of diarrheal diseases are attributed to unsafe water supply, inadequate sanitation, and poor hygiene [5].

In Ethiopia, up to 60% of the current disease burden is attributable to poor sanitation, where 15% of total deaths are from diarrhea, mainly among the large population of children under five years. Children in the country still suffer from diarrheal diseases, respiratory problems, and malnutrition. According to Ethiopian demographic and health survey, the two-week prevalence of diarrheal diseases was 12% among children under five years of age [6, 7].

The local religious institutions are often found at the heart of a community, not only upholding belief, cultural values, and social tradition, but also as a force for positive change and development. Holy springs are frequently contaminated with fecal bacteria, and different infections are potentially transmitted from an infected person to a healthy one by various routes involving excreta [7]. A study from India showed that a possible source of infection for a confirmed case of cholera in a 3-day-old neonate was by holy water given to the baby [8]. Thus, religious institutions might be a point of infection for the community served there, and despite concerted efforts by governmental and nongovernmental organizations, water and adequate sanitation still remain a challenge for these institutions. The study was conducted to determine latrine availability and associated factors among religious institutions in the Tigray Region, Ethiopia, aiming to establish baseline information. This will be very important for local decision makers in order to obtain an overview of the current status and what should be done in the future.

## 2. Methods

### 2.1. Study Design, Setting, and Participants

A cross-sectional, religious institution-based study design was carried out from May to June 2018 in the Tigray Region, Northern Ethiopia, 780 km far from the capital city Addis Ababa. Population source was all religious institutions found in the Tigray Region, while the study populations were those religious institutions found in the selected district.

### 2.2. Sampling Technique and Procedure

Multistage probability sampling of four stages was used to select religious institutions. Using simple random sampling technique, three zones were selected from the seven zones of the Tigray Region, and ten districts were sampled from the selected zones. Then, all the religious institutions found in each district were first listed, and the eligible institutions were included. Thereafter, religious institutions were questioned consecutively till fulfillment of the sample size, which was determined using single population formula with prevalence estimates of 50% and a marginal error of 0.05% at 95% confidence level. The total sample size was 385. Respondents were the heads or delegates of the religious institution, yet in situations where the head or delegate was not available after two or three visits, others in a similar position were questioned, and these were selected purposely.

### 2.3. Data Collection Instrument and Quality Management

The data were collected using face-to-face interviews with the heads of the institutions and observation. One week prior to the actual data collection period, a pretest was done, and based on the findings, minor modifications of questions, wordings, and phrases were made. During the data collection time, a clear introduction explaining the purpose and objectives of the study was provided to respondents. Close supervision, honest communication, and on-spot decisions were done during data collection.

### 2.4. Explanatory Variables

The study variables were selected after reviewing relevant literature [4, 7, 8], according to the objective of the research and considering the local context of the study area. The dependent variable was latrine availability. The independent variables were general characteristics, communication, and behavioral and environmental factors.

### 2.5. Operational Definition

Religious institution: formal institutions which have permanent administration including Christian churches, Muslim mosques, and Catholic churches.

Pretest: pretesting is the stage in survey research when survey questions and questionnaires are tested on members of target population/study population to evaluate the reliability and validity of the survey instruments prior to their final distribution.

Diseased in this institution: this means whether there were any illnesses like diarrhea, related to the environmental sanitation.

### 2.6. Statistical Analyses

Data were coded and entered into EPi-Info version 7 software and analyzed using SPSS version 20. Frequency distribution tables, graphs, and narratives were used to present the findings. Frequency distributions, percentages, and odds ratios (ORs) with 95% confidence level (C.I) were calculated for statistical significance tests between variables, and a logistic regression model was used to identify predictors of latrine availability of religious institutions.

### 2.7. Ethical Consideration

Ethical approval and clearance were obtained from the Tigray Health Research Institution, and official letters were obtained from religious leaders. Written informed consent was warranted from all participants.

## 3. Results

### 3.1. Characteristics of the Religious Institutions

In the present study, a total of 385 religious institutions were sampled and 351 of them were included in the study. Of these, 285 (81.2%) were Orthodox churches. Majority of the religious institutions (288(82%)) were church/mosque and 54% of them (n = 188) were found in rural areas. The mean age of the institution was 83.2 years with ±SD of ±119. The mean age of persons permanently living in the institutions was 11.6 ± 36.65, as members of monasteries and also sometimes students (Table 1).

### 3.2. Environmental Characteristics

Of the institutions, 236 (67.2%) had no latrines. When available, the majority were pit latrines (80, 69.6%). For 57% of the institutions, the reasons for not having latrine were that the cost for building one was too high or neither materials nor external assistance was available. In around 98% of the (*n* = 233) institutions without latrine, the priests and servants defecate openly. At the time of data collection, 82 (23.4%) latrines were functional. Approximately one-third or 41 (35.7%) of the latrines were less than 15 m from the drinking water/holy water. Seventy-six percent of the latrines were at a distance of above 12 m from the room priests serve (Table 2).

### 3.3. Behavioral Characteristics

Regarding the behavioral condition, the majority (319, 90.9%) of the respondents believed that using latrine can prevent disease, and 9 out of 10 respondents believed that hand washing can prevent diseases. Of the religious institutions that have hand washing facilities with a latrine, 32 (82.1%) used only water for hand washing. Fifty-six percent (*n* = 22) of the community or priests serving in the institutions wash their hands after using toilet. Half (*n* = 59, 51.3%) of the respondents who have latrine maintained their latrine properly (Table 3).

### 3.4. Communication-Related Factors

Seventy-eight percent (*n* = 275) of the respondents had seen, heard, or received any messages or materials on sanitation and hygiene. Around one-third of 90 respondents had received messages on building latrine. One hundred twenty five (45.5%) of the respondents heard or observed the messages from community meetings. Three out of ten (*n* = 103, 29.3%) of the sanitation and hygiene messages were delivered by health extension workers. Less than half of 153 (43.6%) respondents prefer radio or/and television for health education (Table 4).

### 3.5. Factors Associated with the Availability of Latrine

To identify significant variables that were associated with the outcome variable, all significant variables with *p* value <0.25 in bivariable analysis were fitted into the final model. Currently, any money saved towards having a latrine (AOR: 0.32, 95% CI [0.25, 0.42]), seen, heard, or received any messages on sanitation and hygiene (AOR: 0.43, 95% CI [0.38, 0.51]), and place where these messages had been seen, heard, or received (AOR: 2.95, 95% CI [1.11, 5.55] were the independent factors of availability of latrine.

Religious institutions that had not saved money for sanitation and hygiene were 68% times less likely to have latrine than institutions that had saved money. The heads of religious institutions that had not seen, heard, or received any messages on sanitation and hygiene were 57% times less likely to have latrine than those who had. The heads of religious institutions that had received messages from posters or leaflets and newspapers or magazines were 2.95 times more likely to own latrine than those having received messages by television and/or radio (Table 5).

## 4. Discussion

The main objective of this study was to assess the level of latrine availability and its associated factors in religious institutions in the Tigray Region. Accordingly, the present study revealed that the overall availability of latrine was 32.8%. The national and regional target for latrine availability is 100% in all settings [9]. However, the study reported that only three out of ten religious institutions had a latrine, implying that there is very low sanitary coverage among religious institutions in the region. This result was low compared with a previous study reporting that 59% of households in Ethiopia own a latrine [10] and the 2016 Ethiopian Demographic Health Service which showed that 56% of rural households use unimproved toilet facilities [11]. This might be due to the fact that there were no persistent health education programs carried out to visitors of religious institutions. Consequently, communities who are served there will not perceive that building latrines can prevent from different diseases and childhood diarrhea. Another possible reason could be that heads of the institution did not handle visitors' defecation practice strongly enough.

This study exposed that religious institutions that had saved money for sanitation and hygiene were significantly associated with availability of latrine. This is consistent with a study carried out by the World Bank [12], which indicates that “those without latrines tend to be poorer than those higher on the sanitation ladder and open defecators cite “lack of finances” or “do not have money” as key barriers to building latrines or making improvements.” Thus, religious institutions that have saved money for the purpose of latrine construction might employ daily laborers to construct the latrine. A study indicated that latrine promotion programs like community-led total sanitation and hygiene were least effective in communities where subsidies had already been given to the community members, meaning that they became reluctant and already expected to get gifts [13]. Thus, institutions having saved money for the purpose of latrine construction might own or build one.

Heads of religious institutions that had not seen, heard, or received any messages on sanitation and hygiene were 57% times less likely to own latrine than those who had, likely because the latter were better informed about the importance of building latrine facilities and its utilization through health-promotion programs and community mobilization. If communities visiting religious institutions get information about basic sanitation, they might perceive the risk of practicing open defecation, which has the potential to stimulate and shape communities' behaviors [14].

Religious institutions having received information from posters or leaflets and newspapers or magazines were more likely to own latrine than those which received messages by television and/or radio. According to this socioeconomic feature of the community, governmental and nongovernmental community education programs should utilize low-cost mechanism of transmissions. Generally, to increase latrine availability, health professionals should sustainably educate on the implementation of the community-led total sanitation and hygiene approach [15].

## 5. Conclusion

The availability of latrine was very low when compared to the national target of 100% among religious institutions, and over half of the available latrines required maintenance. Latrine availability was influenced by the currently saved money towards having this basic sanitation facility, any messages on sanitation and hygiene received, and the place where the message had been received. Information regarding latrine availability should be provided to the community visiting religious institutions through available channels and practical model promotion. Messages focusing on proper disposal of human feces should be scaled up throughout the community. Providing enough information about latrine construction and cleanliness through health education is indispensable to improve latrine availability.

## Figures and Tables

**Table 1:** General characteristics of religious institutions, Northern Ethiopia, 2017 (*n* = 351).

Characteristics	Category	Frequency	Percentage
Type of religious institution	Orthodox church	285	81.2
	Muslim mosque	60	17.1
	Catholic church	4	1.1
	Protestant church	2	0.6

Service given in the institution	Church/mosque only	288	82.1
	Church with holy water	63	17.9

Residence	Urban	159	45.8
	Rural	188	54.2

**Characteristics**		**Mean** **±** **SD**	
Age of the institution		83.2 ± 119	
Age of the respondent		52.3 ± 15.26	
People permanently living here		11.6 ± 36.65	
Estimation of the community served here		1308 ± 3822	

**Table 2:** Environmental conditions of religious institutions, Northern Ethiopia, 2017 (*n* = 351).

Characteristics	Category	Frequency	Percentage
Any type of latrine	Yes	115	32.8
	No	236	67.2

Type of latrine	Pit latrine	80	69.6
	Others	35	30.4

Reasons for not having latrine	Cost is too high, no materials, and no external assistance	202	85.47
	Open defecation tradition and habit	19	8.12
	Not thought about it and no one to build latrine	15	6.41

Place of defecation	Open field	233	98.7
	Other	3	1.3

Functional latrine	Yes	82	71.30
	No	33	28.70

Distance of latrine to the closest drinking water/holy water	Below 15 meters	41	35.7
	15–30 meters	25	21.7
	Greater than 30 meters	49	42.6

Distance of latrine from the room priests serve	Below 6 meters	18	15.7
	6–12 meters	9	7.8
	Above 12 meters	88	76.5

Number of rooms of the latrine	Below 2 rooms	59	51.3
	2–4 rooms	29	25.2
	Above 4 rooms	24	23.5

Clean latrine	Yes	55	47.8
	No	60	52.2

Frequency of cleaning latrine	Daily	42	36.5
	Weekly	35	30.4
	Almost never	38	33.0

Presence of hand washing	Yes	39	33.9
	No	76	66.1

Type of hand washing	Tap only and sink	18	46.2
	Water pot/container and cup	21	53.8

Latrine condition	Need maintenance	80	69.6
	No need of maintenance	35	30.4

Reasons for not improving/changing latrine type	Financial problem/no support	40	50.0
	Personal and space problem	40	50.0

Possible ways encouraging you to build a latrine	Full subsidy and contribution from NGOs	147	62.3
	Community pressure and/or material and labor assistance	89	37.7

Anyone diseased in this institution	Yes	42	12.0
	No	309	88.0

Currently any money saved towards having a latrine	Yes	16	6.8
	No	220	93.2

Institution discussed about building latrine	Yes	113	47.9
	No	123	52.1

**Table 3:** Behavioral conditions of religious institutions, Northern Ethiopia, 2017.

Characteristics	Category	Frequency	Percentage
Do you believe that using latrine can prevent disease?	Yes	319	90.9
	No	32	9.1

Do you believe that hand washing can prevent disease?	Yes	320	91.2
	No	31	8.8

Materials used in hand washing	Only water	32	82.1
	Water + soap or ash	7	17.9

Wash their hands after using toilet (community and priests)	Yes	22	56.4
	No	17	43.6

Maintaining the latrine properly	Yes	59	51.3
	No	56	48.7

**Table 4:** Communication-related factors of religious institutions, Northern Ethiopia, 2017.

Characteristics	Category	Frequency	Percentage
Seen, heard, or received any messages or materials on sanitation and hygiene	Yes	275	78.3
	No	76	21.7

Kinds of sanitation and hygiene messages you have seen, heard, or received	Build a latrine	90	32.8
	Use a latrine/stop open defecation	58	21.2
	Proper solid and liquid waste management	15	5.5
	Wash hands with soap	65	23.7
	Water and food hygiene	46	16.8

Where did you see, hear, or receive these messages	Posters or leaflets and newspapers or magazines	55	20.0
	At community meetings	125	45.5
	When visiting a health facility	56	20.4
	On television and/or radio	39	14.2

From whom did you hear/receive these messages	Village chief	82	29.8
	Commune chief/council	29	10.5
	Government agency other than health department	35	12.7
	From health extension workers	103	29.3
	From coordinators of church	26	7.4

Preferred channel of communication or mechanisms to get information	Radio or/and TV	153	43.6
	House visit	90	25.6
	Through church/mosque	63	17.9
	Pictures/posters	45	12.8

**Table 5:** The main predictors of latrine availability among religious institutions of Tigray Region, Northern Ethiopia, 2018 (*n* = 351).

Characteristics	Latrine availability, *n* (%)		OR (95%CI)	
	Yes	No	Crude	Adjusted
*Possible ways encouraging you to build a latrine*				
Full subsidy and contribution from NGOs	12 (8.16)	135 (91.84)	1.42 (0.58–3.45)	**NS**
Community pressure and/or material and labor assistance	10(11.24)	79 (88.76)	1	

*Currently any money saved towards having a latrine*				
Yes	9 (56.25)	7 (43.75)	1	**1**
No	57 (25.91)	163 (74.09)	0.27 (0.09–0.76)	**0.32** (**0.25**–**0.42**)

*Institution discussed about building latrine*				
Yes	34 (30.09)	79 (69.91)	**1**	**NS**
No	30 (24.39)	93 (75.61)	**0.75** (**0.42**–**1.33**)	

*Seen, heard, or received any messages or materials on sanitation and hygiene*				
Yes	105 (38.18)	170 (61.82)	**1**	**1**
No	10 (13.16)	66 (86.84)	**0.25** (**0.12**–**0.49**)	**0.43** (**0.38**–**0.51**)

*Kinds of sanitation and hygiene messages you have seen, heard, or received*				
Build a latrine	26 (28.89)	64 (71.11)	**1.89** (**0.90**–**3.97**)	NS
Use a latrine/stop open defecation	27 (46.55)	31 (53.45)	**0.88** (**0.41**–**1.92**)	
Proper solid and liquid waste management	8 (53.33)	7 (46.67)	**0.67** (**0.21**–**2.17**)	
Wash hands with soap	24 (36.92)	41 (63.08)	**1.31** (**0.61**–**2.84**)	
Water and food hygiene	20 (43.48)	26 (56.52)	**1**	

*Where did you see, hear, and receive these messages*				
Posters or leaflets and newspapers or magazines	17 (30.91)	38 (69.09)	2.35 (1.01–5.50)	2.95 (1.11–5.55)
At community meetings	48 (38.40)	77 (61.60)	1.69 (0.82–3.48)	1.95 (0.86–3.58)
When visiting a health facility	14 (25.00)	42 (75.00)	3.16 (1.32–7.55)	3.01 (1.31–6.55)
On television and/or radio	20 (51.28)	19 (48.72)	1	1

*From whom did you hear/receive these messages*				
Village chief	23 (28.05)	59 (71.95)	2.99 (1.21–7.43)	**NS**
Commune chief/council	13 (44.83)	16 (55.17)	1.44 (0.49–4.16)	
Government agency other than health department	16 (45.71)	19 (54.29)	1.39 (0.50–3.84)	
From health extension workers	39 (37.86)	64 (62.14)	1.92 (0.80–4.56)	
From coordinators of church	14 (53.85)	12 (46.15)	1	

**Age of the institution**			**1.00** (**1.0**–**1**)	**NS**
**Age of the respondent**			**1.014** (**0.99**–**1.03**)	**NS**
**People permanently living here**			**0.99** (**0.99**–**1.00**)	**NS**

NS indicates nonsignificance.

## Data Availability

The datasets used and/or analyzed during the current study are available from the corresponding author on request.

## Abbreviations

AOR: Adjusted odds ratio
CI: Confidence interval
DALY: Disability-adjusted life year.
